# Overcoming the obstacles of current photodynamic therapy in tumors using nanoparticles

**DOI:** 10.1016/j.bioactmat.2021.06.019

**Published:** 2021-06-26

**Authors:** Donghyun Lee, Soonmin Kwon, Seok-young Jang, Eunyoung Park, Yeeun Lee, Heebeom Koo

**Affiliations:** Department of Medical Life Sciences, Department of Biomedicine & Health Sciences, and Catholic Photomedicine Research Institute, College of Medicine, The Catholic University of Korea, 222 Banpo-daero, Seocho-gu, Seoul, 06591, Republic of Korea

**Keywords:** Photodynamic therapy, Nanoparticle, Tumor-targeting, Drug delivery, Tissue penetration

## Abstract

Photodynamic therapy (PDT) has been applied in clinical treatment of tumors for a long time. However, insufficient supply of pivotal factors including photosensitizer (PS), light, and oxygen in tumor tissue dramatically reduces the therapeutic efficacy of PDT. Nanoparticles have received an influx of attention as drug carriers, and recent studies have demonstrated their promising potential to overcome the obstacles of PDT in tumor tissue. Physicochemical optimization for passive targeting, ligand modification for active targeting, and stimuli-responsive release achieved efficient delivery of PS to tumor tissue. Various trials using upconversion NPs, two-photon lasers, X-rays, and bioluminescence have provided clues for efficient methods of light delivery to deep tissue. Attempts have been made to overcome unfavorable tumor microenvironments via artificial oxygen generation, Fenton reaction, and combination with other chemical drugs. In this review, we introduce these creative approaches to addressing the hurdles facing PDT in tumors. In particular, the studies that have been validated in animal experiments are preferred in this review over proof-of-concept studies that were only performed in cells.

## Highlights

• Successful photodynamic therapy in tumors has been inhibited by obstacles including insufficient amount of photosensitizer, light attenuation, and unfavorable tumor microenvironmrnt.
• Until now, various nanoparticles have been developed and used for tumor-targeted photodynamic therapy.
• The nanpparticles showed potential to overcome the obstacles by efficient supply of photosensitizers, light, and oxygen.

## 1 Introduction

Photodynamic therapy (PDT) is a clinical treatment using a laser and photosensitizer (PS), which is a special kind of fluorescent dye. When irradiated with intense light, the PS transfers energy to surrounding oxygens, generating reactive oxygen species (ROS) that kill the target cells [1]. It is highly useful in cases where people want to kill specific cells or destroy tissues like cancers. PDT has been widely applied in clinical treatment of various cancers (e.g., prostate, breast, head and neck, skin, pancreas, and lung) [2,3]. To increase water solubility of the PS and improve its delivery efficiency, nanoparticles (NPs) have been used as promising carriers [4]. For efficient delivery of PSs for PDT, various kinds of NPs have been developed using different materials such as polymer, lipid, gold, silica, carbon, and iron oxide, which have shown promising results in many papers [[5], [6], [7]].

PSs have been physically loaded or chemically conjugated to NPs for their delivery, and each method has pros and cons [8]. Generally, physical loading has been used in self-assembled organic NPs, which have an amphiphilic structure. Because many PSs are hydrophobic, they are localized in hydrophobic pockets of self-assembled NPs. In this case, the stability and release pattern of the loaded PSs are dependent on hydrophobic interactions, so that the relative hydrophobicity of the PS has a great influence. In our recent paper, two kinds of PSs, chlorin e6 (Ce6) and pheophorbide a (Pba) were separately loaded in poly (lactic-*co*-glycolic acid) (PLGA) NPs [9]. The chemical structures of Ce6 and Pba are similar, but Ce6 is more hydrophilic than Pba. After intravenous injection of these NPs into tumor-bearing mice, Pba was more efficiently delivered to tumor tissue by NPs due to stable loading and reduced burst release in blood. These results demonstrated that the hydrophobicity of PSs should be considered carefully when they are physically loaded into NPs. Chemical conjugation is not preferred over other loading methods in general drug delivery because the therapeutic efficacy of most chemical drugs is highly reduced when they are conjugated to other molecules. However, it has been reported that PSs can generate ROS whether or not they are conjugated, which means that they are still active after conjugation. Therefore, many studies have used chemical conjugation for PS delivery by NPs. In addition, chemical conjugation can prevent unintended release of PSs during blood circulation, which is helpful to enhance their effectiveness at targeting tumors. However, conjugated PSs resulted in slow excretion from the body due to the increased molecular weight, so that unintended phototoxicity in normal tissue should be considered carefully from a long-term perspective.

**Scheme 1:**
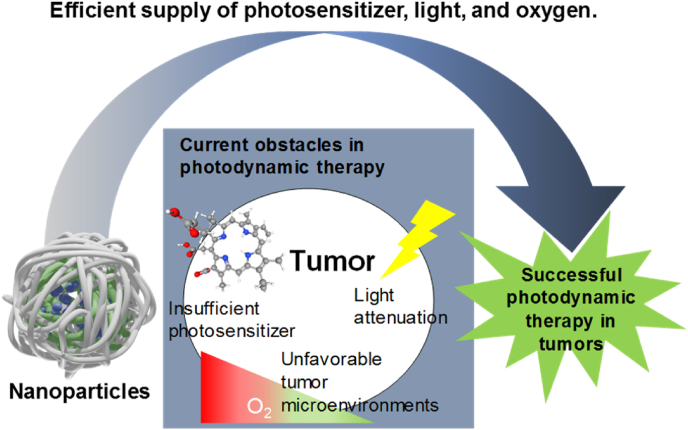
Schematic illustration of three major obstacles in current photodynamic therapy in tumors and use of nanoparticles to overcome them.

To date, many NPs have been developed for PDT, and their effectiveness has been demonstrated in animal studies, but PDT still faces many obstacles that limit its broader application. Basically, three essential factors in PDT are PS, light, and oxygen [10]. Therefore, if one of these factors is not supplied sufficiently at the target disease site, the therapeutic efficacy of PDT will be reduced. In this review, we focused on the representative obstacles of PDT: inefficient PS delivery, poor light penetration to deep tissue, and hypoxia in tumor. We introduce cutting-edge researches based on rationally designed NPs to overcome these obstacles (Scheme 1). Combination of nano- and biotechnology resulted in many promising NPs that have shown clues about how to overcome these obstacles of current PDT. In particular, the studies that were validated in animal experiments are preferred in this review over proof-of-concept studies that were only performed in cells. Remarkable progress in new delivery systems in recent PDT has been summarized well in many previous reviews [[11], [12], [13], [14]]. However, most of them relatively focused on the materials themselves, but our review first looked at the obstacles more carefully and then tried to focus on the recent promising efforts to solve them.

## 2 Efficient photosensitizer delivery

Efficient delivery of PS to the target tissue and cells has been the most basic function of NPs in PDT [15]. Traditionally, the size or surface properties of NPs have been optimized for passive targeting based on the enhanced permeability and retention (EPR) effect [16,17]. To enhance specificity, various ligands binding cell surface receptors have been attached to the surface of NPs for active targeting [18]. Focused on the stimuli-responsive systems, researchers also realized controlled changes of NP structure or triggered release of PSs in tumor tissue [19].

### 2.1 Passive targeting and its enhancement

Tumor vascular tissue has a leaky endothelial cell layer and insufficient lymphatic drainage due to rapid neovascularization associated with rapid tumor growth. In contrast, endothelial cells in normal vessels are too dense for NPs to extravasate. Therefore, intravenously administrated NPs accumulate more in tumor tissue than in normal tissue, which is a main story of traditional EPR effect [17]. To maximize this effect, physicochemical properties of NPs including size, charge, and shape should be optimized [20,21]. Tumor targeting strategies of NPs for PDT are similar to those of NPs for traditional drug delivery, and they are well-summarized in other review papers [16]. Additionally, Huang group introduced human serum albumin (HSA) NP loading paclitaxel and sinoporphyrin as PS [22]. Albumin is a good candidate as drug carrier because of its long half-life and continuous uptake in tumor tissue, which could enhance EPR effect. They also tried to increase the delivery efficiency of HSA NPs by perturbation of cell membrane with low-intensity ultrasound (US). During *in vitro* study, the fluorescence intensity of 4T1 cells treated with the HSA-NPs increased approximately 1.47 times due to the increased cellular uptake by cell membrane perturbation after US treatment. The researchers established mice model with 4T1 tumor on both thighs. They injected the HSA-NPs intravenously into the model and US was irradiated in only one tumor to investigate the increased tumor accumulation of HSA-NPs. As a result, US-irradiated tumor showed significantly higher amount of HSA-NP compared to non-irradiated tumor. After PDT, tumor growth inhibition rate was 92.4% in both US and laser-treated mice but 76% in laser-only group by enhanced EPR effect due to US-induced tumor accumulation of NPs.

**Fig. 1:**
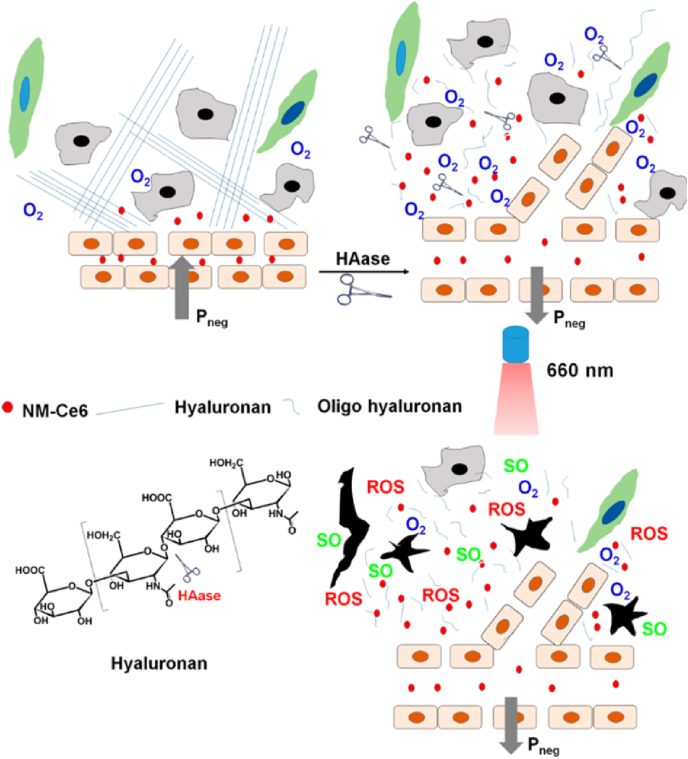
Hypoxia-responsive dissociation of nanoparticles for deep tissue penetration and photodynamic therapy. Adapted from Ref. [23]. Copyright 2016 American Chemical Society.

To maximize the EPR effect and increase the tumor-targeting of NPs in passive targeting, efficient penetration into tumor tissue through extracellular matrix (ECM) is important. In this way, Gong et al. improved tumor targeting of their NPs by injecting hyaluronidase (HAase) into the tumor site to decompose the ECM structure (Fig. 1) [23]. HA is a major component of ECM, and it can be degraded by HAase. When HA in ECM breaks down, the interstitial flow pressure in the tumor is decreased, which can lead to normalization of the tumor vessel structure, increasing the accumulation of NPs in the tumor tissue overall. To demonstrate this effect, the authors intratumorally injected HAase into 4T1 tumor-bearing BALB/c mice and then performed ultrasound imaging. They found that vascular perfusion was increased in the group injected with HAase compared to the group injected with saline intratumorally. Then the authors fabricated PEGylated and chlorin e6 (Ce6)-conjugated polymeric micelle that was 33 nm in size. To analyze the effect of HAase treatment upon the EPR effect-based tumor targeting of NPs, they prepared BALB/c mice with 4T1 tumors on both thighs. When only one tumor was treated with HAase and the NPs were injected intravenously, approximately twice as many NPs accumulated in tumors injected with HAase compared to non-treated tumors due to enhanced EPR effect due to the degradation of ECM structure by HAase. Furthermore, PDT was performed in the same mice models to show improvement of therapeutic efficacy by administration of HAase. The group injected with HAase alone could not suppress the tumor. The group treated with HAase and NPs showed stronger tumor suppression ability than the group injected with NPs alone. This means that enhanced tissue penetration by ECM degradation with HAase can contribute to improving tumor-targeting of NPs and the efficacy of PDT treatment.

### 2.2 Stimuli-responsive changes

Microenvironments in tumor tissue or cells are different from those in normal tissue, which can be a stimulus for drug release from NPs. Rationally designed chemical structures and components in NPs can provide stimuli-responsive properties after administration into the body [24]. These types of NPs can contain drugs stably during blood circulation and release them or activate them after they reach target tumor tissue or cells in response to the changes in various stimuli, including both internal (pH, enzymes, ATP, and oxygen concentration) and external ones (light, heat, magnetic field and ultrasound) [25]. Among them, hypoxia is one of the representative special situations in tumor tissue. Hypoxia occurs due to the characteristics of aggressively growing cancer cells, including unlimited multiplication, vigorous energy consumption, and insufficient oxygen supply [26].

**Fig. 2:**
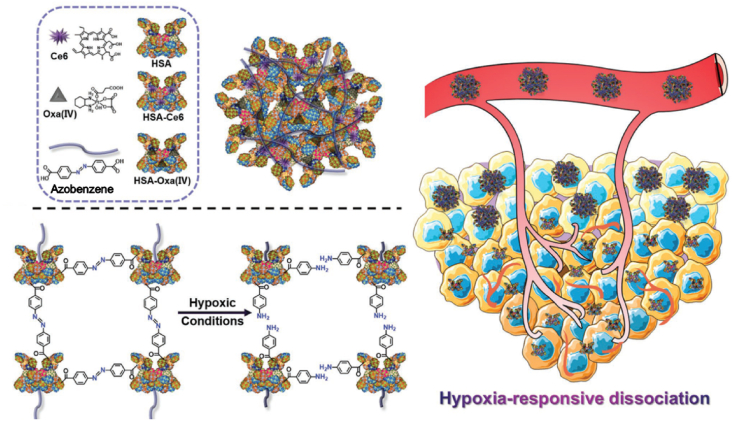
Precise mitochondria targeting after pH-responsive dissociation of nanoparticles for photodynamic therapy. Adapted from Ref. [27]. Copyright 2020 Springer.

Focused on this, Yang et al. developed hypoxia-responsive NPs based on human serum albumin (HSA) for PDT [27]. The authors crosslinked chlorin e6 (Ce6)-conjugated HSAs and oxaliplatin prodrug-conjugated HSAs via hypoxia-sensitive azobenzene group (Fig. 2). These NPs are stable, with a size of 128 nm, under normoxia. However, they can dissociate quickly into ultrasmall HSAs containing Ce6 or prodrugs with diameters of below 10 nm when exposed to a hypoxic microenvironment, which can enhance tumor penetration of NPs. Importantly, they can recover fluorescence for imaging and PDT ability after dissociation because of escaping from an aggregated state. In addition, the conjugated oxaloplatin prodrugs can further increase antitumor efficacy by combinational therapy. During *in vitro* experiments, the authors evaluated these advantages of hypoxia-responsive NPs using the 4T1 cell line in normoxia or hypoxia. They showed that these NPs killed cancer cells with 660-nm laser irradiation more effectively under both normoxia and hypoxia compared to control groups with PDT or chemotherapy alone. Then, they injected the NPs in 4T1 tumor-bearing Balb/c mice intravenously and irradiated the tumor site with a 660-nm laser. The treatment showed 4.5-fold higher antitumor efficacy than the control group. These results may be due to the improved deep tumor penetration provided by the hypoxia-responsive disassociation of NPs, selectively increased singlet oxygen production, and effective combination of PDT and chemotherapy.

Another study using a ROS-responsive linker for controlled release of PSs was published by the Zhang group [28]. The authors used vinyldithioether as a cleavable linker to connect amphiphilic block copolymer and hydrophobic pyropheophorbide a (Ppa), a PS. Owing to the aggregation of hydrophobic segments, the amphiphilic block copolymer formed NPs in aqueous solution through self-assembly. The singlet oxygen-responsive vinyldithioether linker can selectively release Ppa through degradation by producing ROS after a short period of laser irradiation. They injected the NPs into A375 human malignant melanoma tumor-bearing BALB/c nude mice via a tail vein. Twenty-four hours post-injection, the tumor site was irradiated with a 660-nm laser for 1 min to selectively release PSs from cleaved vinyldithioether. After that, the secondary laser irradiation was performed for PDT effect at intervals of 4 h. These self-amplified NPs displayed significant inhibition of tumor growth under observation for 21 days, compared with the control group without cleavable linkers or second laser irradiation. This is because the enhanced therapeutic photoactivity by accelerated release of Ppa.

### 2.3 Active targeting

**Fig. 3:**
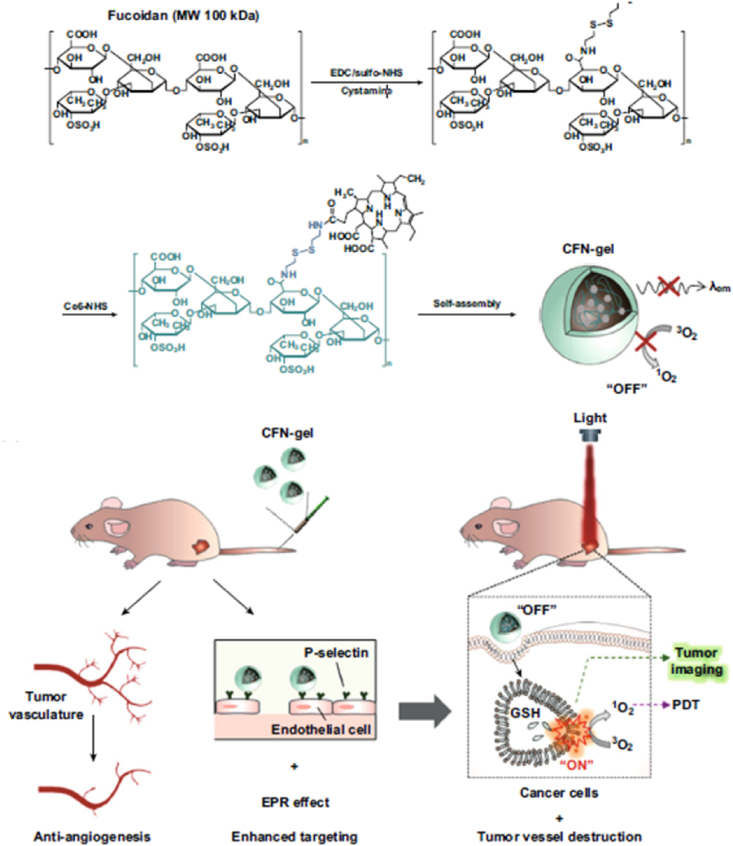
Active targeting against P-selectin on endothelial cells in tumor tissue with fucoidan nanoparticles for photodynamic therapy. Adapted from Ref. [30]. Copyright 2019 WILEY-VCH.

For active targeting, the surface of the NP has been modified with biological ligands with affinity for specific receptors on target cells [29]. Antibodies, aptamers, peptides, and small molecules have been used for active targeting of NPs. If a NP is developed using a polymer that has a binding affinity to receptors, it can be used as a shell component of the NP and active targeting ligand simultaneously, as shown in the recent work by the Choi group [30]. The authors used fucoidan, a biopolymer from seaweeds. Fucoidan can bind to P-selectin, which is overexpressed on neovascular endothelial cell surfaces in many cancers (Fig. 3). Also, fucoidan has negative charges that prevent aggregation with cationic proteins in blood. Furthermore, fucoidan is reported to have an anticancer effect by controlling a scavenger receptor, blocking metastasis, and activating immune function. Disulfide linkers were used to fabricate a Ce6-fucoidan polymer for selective degradation in tumor cell cytosol after uptake. The Ce6-fucoidan polymer conjugate formed nanogels via self-assembly because of aggregation of hydrophobic Ce6 molecule. In HT1080 fibrosarcoma tumor-bearing BALB/c nude mice, these fucoidan nanogels showed more than 10 times higher tumor accumulation of Ce6 compared to free Ce6 24 h post-injection by tail vein due to EPR effect and active targeting ability of fucoidan to P-selectin. Upon laser irradiation, the NPs showed complete tumor suppression with effective apoptosis by PDT.

ROS produced by PDT cannot damage biomolecules from a distance due to their short lifetime and great reactivity. Therefore, delivering PS to essential organelles such as mitochondria in cancer cells would be advantageous for efficient PDT. A mitochondria-targeting strategy is needed after cellular uptake, so that other strategies such as stimuli-responsive changes are applied until reaching cytosol in general. For example, Qi et al. fabricated NPs containing triphenyl phosphonium (TPP)-conjugated mitochondria-targeting PS (TPP-PS) and pH-responsive polymer labeled with Cy7.5 [31]. At pH 7.4, fluorescence of the TPP-PS loaded in the NPs was quenched because of hetero-fluorescence resonance energy transfer (hetero-FRET). However, it increased 107-fold below pH 6.4, and singlet oxygen generation was also recovered by decomposition of the NPs. It could reduce non-specific toxicity of TPP-PS in normal tissues. In HO8910 human ovarian cancer cells, the authors verified the mitochondria-targeting ability of the NPs using a confocal laser scanning microscope (CLSM). TPP-PS was colocalized with mitochondria in 30 min, but the backbone of NPs and TPP-PS were separated inside the cells. This indicated that the TPP-PS was rapidly released from the NPs by acidic endosomal conditions and then accumulated in mitochondria. The *in vitro* cytotoxicity of the NPs containing TPP-PS under 660-nm laser irradiation was higher in HO8910 cells than that of free PS due to their mitochondria targeting ability. To evaluate the antitumor effect of NPs, the experimental groups were injected intravenously into HO8910 tumor-bearing nude mice. Results indicated that mitochondria-targeting TPP-PS improved antitumor efficacy relative to the control PS. Furthermore, the TPP-PS-containing NPs showed better antitumor effect than free TPP-PS because of the EPR effect, which was enough to eliminate half of the tumors at 33 days after treatment. These results demonstrate that mitochondria-targeting of PS is effective for enhancing PDT efficacy.

TPP group with cationic charges increases membrane binding and mitochondria targeting, but it is difficult to use *in vivo* as itself because of rapid clearance in blood circulation. To overcome this problem, the Liu group developed pH-controlled charge reversal micelle, which can change anionic charges to cationic charges in acidic tumor extracellular matrix (ECM) [32]. The micelle core was composed of Ce6 loaded micelle with positive charge containing TPP, and it was coated with pH-sensitive 2,3-dimethylmaleic anhydride (DMA) polymer as a shell. In the proposed sequential-targeting mechanism, the first tumor cell targeting is achieved by biotin moiety on the shell of the micelles. Then, in the second step, mitochondria are targeted by TPP moiety of core micelle after hydrolysis of the amide bond in DMA in acidic tumor ECM. The charge-reversible micelle showed 3.7-fold higher accumulation in tumor tissue compared to irreversible micelle because positively charged micelle could be favorable to be trapped within tumor. The micelle showed significant suppression of tumor volume for 14 days via intravenous injection in CT26 tumor-bearing BALB/c nude mice. Compared to the group without TPP or DMA, the micelle showed about three times-enhanced anticancer effect. Their strategy enhanced the PDT effect by targeting tumor cells and mitochondria simultaneously and provided the inspiration to solve the problem during *in vivo* delivery of cationic NPs.

## 3 Light delivery to deep tissue

PDT is based on light irradiation and stimulation, so that light should be delivered to the target cells for efficient PDT. However, the intensity of light is reduced quickly while penetrating tissues due to absorption by collagen, water, and other biomolecules. When X-ray penetrates 3 cm thick tissue, attenuation of about 50% occurs, whereas the visible light area shows much lower transmittance. The tissue penetration depth of UV, blue, green, yellow, red, NIR was reported as about 0.1, 0.3, 0.5, 1, 2 and 3 mm respectively [33,34]. The tissue penetration depth increases with longer wavelength in visible light area. Therefore, short wavelength-light cannot be delivered to deep tissue, which may result in incomplete PDT in tumor and regrowth. To overcome the light attenuation in tissue, researchers have tried various methods including upconversion NPs, two-photon lasers, X-rays, and bioluminescence.

### 3.1 Upconversion nanoparticles (UCNPs)

Upconversion nanoparticles (UCNPs) are nanoscale materials that can enable photon upconversion. During this process, the NPs absorb two or more incident photons having relatively low energy and convert them into a single photon emitted with higher energy. This means that, unlike general fluorescent dyes, they can absorb near-infrared red (NIR) light (longer wavelength) and emit ultraviolet (UV)/visible light (shorter wavelength), [35]. UCNPs usually consist of rare-earth-based lanthanide- or actinide-doped transition metals. Interest has grown in potential biomedical applications such as imaging and therapy because of superior optical penetrating ability through deep tissue with little background noise. When UCNPs are applied in PDT, they can allow deeper-penetrating NIR light (generally from 800 to 1000 nm), which has a longer wavelength than the intrinsic absorbance range of traditional PSs for activation.

Recently, Li et al. developed pH-sensitive NPs consisting of UCNPs, PSs (Ce6), and pH-responsive polymeric ligands [36]. At pH 7.4, The NPs are negatively charged, but can change their surface charge to a positive charge at pH 6.5 (the pH of tumor microenvironments). They are further dissociated into individual UCNPs at pH 5.5 (lysosomal pH) and help the disassembly of the aggregated PSs into extended free molecules enhancing photoactivity. Upon irradiation with a 980-nm NIR laser, which can reach deeper tissue, UCNPs can induce the excitation of the free PSs by emitting upconverted light even after penetration through 7-mm-thick porcine tissue. After intratumoral injection into A549 tumor-bearing BALB/c nude mice, they exhibited even higher antitumor efficacy in response to 980-nm NIR laser than with the red light laser with shorter wavelength that is traditionally used.

**Fig. 4:**
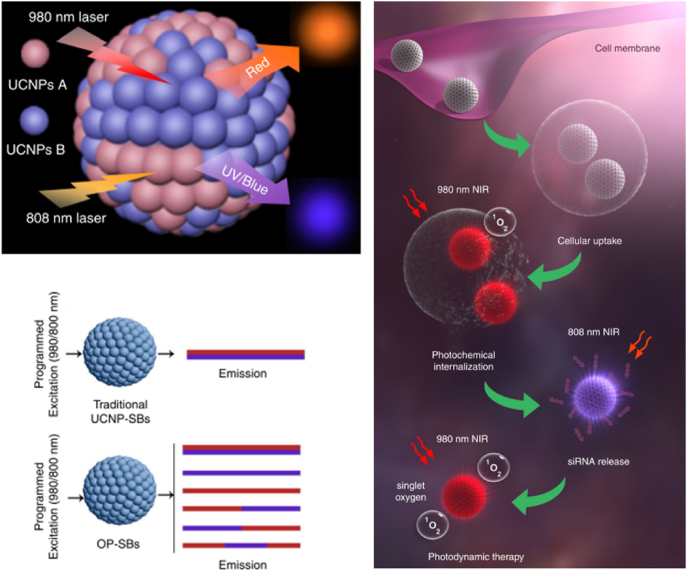
Upconversion superballs for sequential siRNA delivery and photodynamic therapy by infrared laser irradiation. Adapted from Ref. [37]. Copyright 2019 Springer Nature.

Another interesting study using UCNPs was introduced by the Zhang group [37]. The authors developed orthogonal photoactivable-superballs (OP-SBs), which were made of two different UCNPs excited at 980 and 808 nm, respectively (Fig. 4). Consequently, they can release different wavelengths of upconverted light upon irradiation at 980 or 808 nm, which means that they have two kinds of orthogonal triggers for different functions. OP-SBs contain zinc phthalocyanine (ZnPc), a PS for photochemical internalization (PCI)/PDT, and superoxide dismutase 1 (SOD1) siRNA with UV light-responsive linker. After cellular uptake, OP-SBs have red emission and activate PCI under 980-nm irradiation for a short time, which enables endosomal escape of OP-SBs. Then, 808-nm irradiation generates blue/UV emission from OP-SBs and releases siRNA by stimulating azobenzene-based light activated caps. The released siRNA knockdown SOD1 gene decreases inherent resistance against free radicals. Finally, long-duration irradiation at 980 nm results in full-fledged PDT. During *in vivo* tumor therapy, NPs were injected into Cal-27 tumor-bearing BALB/c nude mice intratumorally, and NPs were sequentially activated with 980- or 808-nm laser at specific time points. This programmed multi-step photoactivation showed better therapeutic results of PDT than simultaneous activation.

### 3.2 Two-photon

**Fig. 5:**
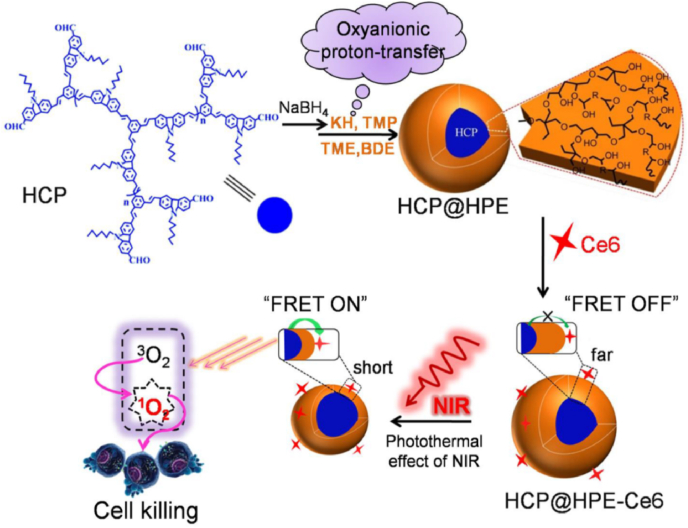
Two-photon-activated fluorescence resonance energy transfer to enhance the efficacy of photodynamic therapy. Adapted from Ref. [39]. Copyright 2017 American Chemical Society.

The efficacy of light delivery to deep tissues is highly dependent upon the wavelength of light. In general, lasers in the NIR wavelength range are known to be more suitable for penetration of deep tissues compared to visible lasers. However, most PSs used in clinics are activated by red light, which is an obstacle for efficient PDT [38]. In this respect, two-photon lasers could be an alternative due to the two-photon absorption phenomenon, which can excite fluorophores using longer-wavelength light. The recent study of Huang et al. is a good example of two-photon-based PDT [39]. They synthesized hyperbranched conjugated polymers with multiple benzene rings for two-photon absorption and coated them with thermo-responsive a hyperbranched polyether shell (Fig. 5). After further adding Ce6 as PS, they fabricated thermo-responsive polymer NPs for two-photon-based PDT. When an 800-nm two-photon laser was applied to the NPs, two-photon fluorescence was observed with a peak at 453 nm, which can excite PS via fluorescence resonance energy transfer (FRET). However, this FRET process only occurs above 39 °C when the distance between 2-PA and PS is shortened due to the phase transition of the polymer by heat after laser irradiation. To demonstrate this effect, *in vitro* phototoxicity assay was performed under irradiation of an 800-nm laser using HeLa cells at different temperatures. Results indicated that the IC50 value at 37 °C was 12.32 μg/ml, whereas at 40 °C, it dropped sharply to 3.95 μg/ml. The authors injected the NPs intravenously into HeLa tumor-bearing mice and significantly suppressed the tumor growth with an inhibition rate of 87.1% by 800-nm laser irradiation with two-photon effect.

Another group was also interested in the merits of two-photon activation such as penetrating deep tissue and entirely preserving one-photon absorption. In order to increase the PDT effect with these merits, the Wang group reported that their water-dispersible polymer NPs could be used for two-photon PDT [40]. They synthesized polythiophene quaternary ammonium (PT2) with pi-conjugated polymer backbone as a two-photon agent via one-pot synthesis. It has two-photon absorption and singlet oxygen generating ability simultaneously. Then, they prepared NPs composed of PT2 and DSPE-PEG 2000 through ultrasonication. The NPs showed a two-photon fluorescence imaging depth of 2100 μm, which was even deeper than the one-photon depth of 800 μm in mock tissue. After intratumoral injection and irradiation with an 800-nm femtosecond laser, they showed a significant therapeutic effect in HeLa tumor-bearing nude mice. These results demonstrated that the resulting NPs provided strong fluorescence of two-photon excited PS, efficient singlet oxygen production, selective lysosome accumulation in cancer cells, and deep tissue penetration.

### 3.3 X-ray PDT

X-rays can penetrate biological tissue much better than visible light, which is a useful characteristic for treating deep-seated tumors. Therefore, the usage of X-rays for PDT will be a promising approach to overcome the problem of penetration depth in PDT. However, most PSs cannot be excited by X-ray, so that energy transducers called scintillators are needed. Scintillators convert X-rays into light that can excite PSs; this event is called X-ray excited optical luminescence. The scintillator was first introduced in 2006, and many researchers have tried to increase the conversion efficiency of scintillators for successful X-ray-activated photodynamic therapy (X-PDT) [41,42].

As a recent example of X-PDT, Yu et al. introduced NPs including Gd_2_(WO_4_)_3_:Tb and merocyanine 540 as a scintillator and PS, respectively [43]. Merocyanine 540 was loaded onto the scintillator. When this scintillator was irradiated with X-rays, it emitted luminescence at about 540 nm, which can excite merocyanine 540. To evaluate X-PDT efficacy, the authors analyzed singlet oxygen generation from the resulting NPs using singlet oxygen sensor green. Among the samples tested, only the resulting NP showed singlet oxygen generation upon X-ray irradiation due to X-PDT effect, while the scintillator or merocyanine 540 alone did not. Then, they compared the cytotoxicity of the resulting NPs with or without PS under X-ray irradiation using the 4T1 cell line. Without PS, the cell viability was 72.3% due to the radiotherapeutic effect of the scintillator only. However, in the presence of PS, it decreased to 54.2% because of the additional X-PDT effect. In 4T1 tumor-bearing mice, the additional functions of the NPs as CT and T1-weighted MRI contrast agent were evaluated. Furthermore, they showed stronger CT and MRI imaging ability than traditional agents such as Iohexol and Gd-DTPA, respectively. In order to evaluate the anticancer efficacy of X-PDT *in vivo*, the authors injected the resulting NPs into 4T1 tumor-bearing mice intravenously, and applied X-rays (6 Gy) to the tumor site. They measured changes in the tumor size for 20 days. On day 20, the relative volume of tumors treated with the NP was 7.9 without PS and 4.3 with PS. These results indicated that X-PDT effect of the NPs also worked well under *in vivo* conditions.

**Fig. 6:**
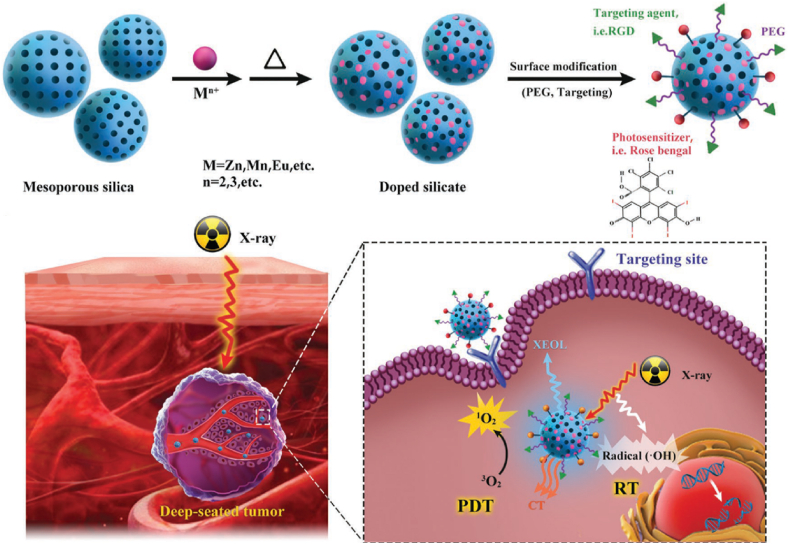
X-ray-induced deep-tissue-penetrating photodynamic therapy using mesoporous silicate nanosensitizers. Adapted from Ref. [44]. Copyright 2017 WILEY-VCH.

As another recent example of X-ray-based PDT, the Chen group's study introduced silicate nanoscintillators composed of Zn, Mn, rose bengal (RB), and arginylglycylaspartic acid (RGD) peptide that enable targeted imaging and PDT [44]. Nanoscintillators were used as transducers converting low-dose X-rays into visible light (peak at 570 nm), overlapping the absorption spectrum of RB (the 480-550-nm absorption band), so that RB could generate singlet oxygen for PDT (Fig. 6). In addition, RB also can absorb X-ray energy due to four iodine atoms in its chemical structure allowing computer tomography imaging and enhanced radiotherapy. This study showed that the developed silicate nanoscintillators could be accumulated in tumors and significantly inhibit tumor progression under low‐dose X‐ray irradiation, while minimally affecting normal tissues. The authors incubated U87MG cells with these NPs and observed significant cancer cell death with X-ray irradiation, while it was not observed without X-ray irradiation. After that, they injected the NPs into U87MG tumor-bearing BALB/c nude mice intravenously and treated them with X-ray irradiation. They achieved 98.1% tumor inhibition rate compared to the control group. Importantly, this treatment worked successfully even when a 1-cm-thick pork tissue block was placed between the X-ray source and tumor, and provided similar therapeutic outcome demonstrating the advantages of X-ray-based PDT for deep tumor penetration.

### 3.4 Chemiluminescence resonance energy transfer (CRET)

**Fig. 7:**
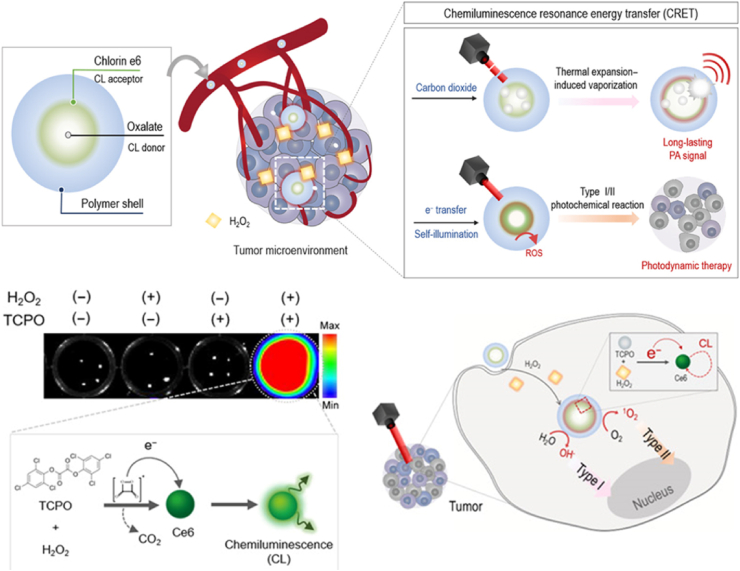
Chemiluminescence resonance energy transfer in self-illuminating nanoparticles for light-less photodynamic therapy. Adapted from Ref. [46]. Copyright 2020 AAAS.

Chemiluminescence resonance energy transfer (CRET) can compensate for light deficiency in deep tissue. Because a chemiluminescent donor transfers energy to an acceptor, CRET does not need an external radiation source for excitation [45]. However, the short duration of the CRET signal resulted in difficulty with diagnosis and insufficient ROS yield in PDT. The recently suggested NP-based CRET system showed effective ROS quantum yield for PDT through close packing of CRET donors and acceptors in NP structure. Jeon et al. constructed self-assembled theranostic CRET-NPs, which consisted of chlorin e6 (Ce6)-conjugated PEG-carboxymethyl dextran (PEG-CMD-Ce6) and bis [3,4,6-tricholoro2-(pentyloxycarbonyl)phenyl]oxalate (TCPO) as CRET acceptor and donor, respectively [46]. The oxidization of TCPO occurs specifically at the ROS-enriched tumor, and produced carbon dioxide (CO_2_), which led to thermal-expansion-induced-vaporization upon pulsed laser irradiation, generating a photoacoustic imaging signal for diagnosis as an additional function (Fig. 7). In the absence of laser irradiation, increased ROS yield was achieved by type 1 oxygen-independent PDT through electron transfer from TCPO to Ce6, and it further increased by type 2 oxygen-dependent PDT after laser irradiation. In HT29 tumor xenograft mice, CRET-NPs showed significant tumor growth suppression without laser irradiation, but it was not complete. These results demonstrated the potential of CRET-based PDT, but further study is needed for clinical application.

Xu et al. also utilized CRET to overcome the limitations of PDT [47]. They synthesized an amphiphilic polymer that is composed of luminol, chlorin e6 (Ce6), and PEG. Luminol, which emits blue light when oxidized, is used as a luminescent donor, and Ce6 acts as a fluorescent acceptor. The luminescence spectrum of luminol at 440 nm overlaps with the absorption spectrum of Ce6 (maximum peak at 401 nm), and as a result, an emission peak at 675 nm from Ce6 can be generated by CRET. The polymer has an amphiphilic feature resulting in a self-assembled NP for *in vivo* luminescence imaging and deep tissue PDT. When these Ce6-luminol-PEG NPs exist in inflammatory sites or tumors, they can produce singlet oxygen by CRET in response to relatively abundant ROS and myeloperoxidase oxidizing luminol compared to other normal tissues. The authors evaluated the utility of these NPs as *in vivo* imaging probes for inflammation diseases such as peritonitis, ulcerative colitis, and acute liver injury in mice models by intraperitoneal injection, intravenous injection, and enema administration. During PDT, they found that these NPs revealed high antitumor efficiency in ROS-abundant cancer cell lines like A549. They injected the NPs into A549 tumor-bearing Balb/c nude mice intratumorally and intravenously without any external irradiation. These treatments could not completely inhibit tumor growth but showed approximately 2-fold higher antitumor efficacy than the control group. These results may originate from insufficient ROS levels in the tumor site for perfect therapy, which will be a challenge for this type of approach.

Bioluminescence from firefly luciferase also showed the possibility of light-free PDT as reported by the Wu group [48]. The authors constructed PLGA NPs loaded with RB. Then, firefly luciferase enzymes were conjugated to the surface of NPs. They can produce bioluminescence in the presence of their substrate luciferin without external light irradiation. The bioluminescence can activate the PS to generate singlet oxygen by bioluminescence resonance energy transfer (BRET), because RB has an absorption band at 480–550 nm, which overlaps with the bioluminescence spectrum of the luciferase-luciferin reaction. In animal experiments, the H22 tumor-bearing mouse was treated with intratumoral injection of the NPs. The therapeutic results showed that the BRET-PDT group was slightly better than the external light excitation group, and the tumor volume was decreased by approximately 6-fold compared to the control group without treatment. The study generated light by luciferase-luciferin reaction, so that it is free from the light-penetration limitation. However, the enzymes on the surface of NPs are not advantageous for anti-fouling effect in blood and tumor targeting, which may be the reason that the authors used intratumoral injection, not intravenous.

## 4 Unfavorable tumor microenvironments

Generation of ROS by PSs upon light irradiation is the basic mechanism of PDT, so its efficacy is dependent on the surrounding oxygen molecules. Unfortunately, the oxygen concentration in tumor tissue is generally lower than the normal state due to hypoxia originating from the rapid outgrowth of the tumor and following insufficient blood supply [49]. This means that continuous maintenance of the oxygen concentration is also an important factor for PDT in tumor tissue. For this reason, researchers have tried to induce oxygen generation artificially or reduce oxygen consumption [50]. Oxygen-independent reactions like the Fenton reaction and synergetic combination with hypoxia-related chemical drugs also have shown promising results.

### 4.1 Oxygen generation or reduced consumption

**Fig. 8:**
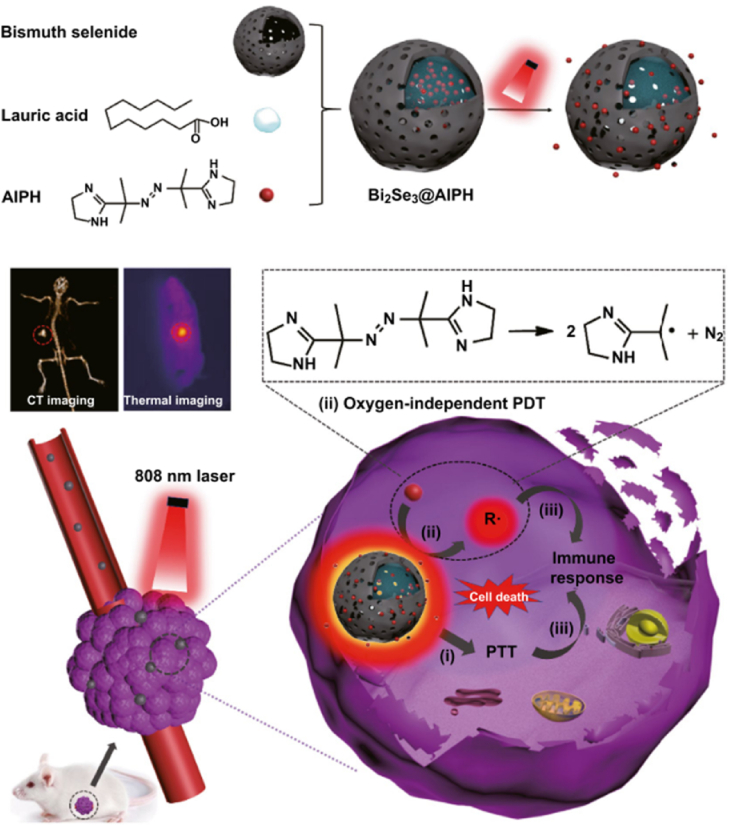
Oxygen-independent photodynamic therapy using bismuth selenide nanoparticles containing free radical generator. Adapted from Ref. [51]. Copyright 2019 Springer.

Many researchers have reported combining PDT and photothermal therapy (PTT) with synergistic effect, but hypoxic condition in tumor is still a problem in these cases. To solve this problem, Li. et al. introduced bismuth selenide NPs that were used for PDT, PTT, and computed tomography (CT) imaging [51]. They contained oxygen-independent radical generator (2,2-azobis [2-(2-imidazolin2-yl) propane] dihydrochloride (AIPH)) and thermo-sensitive lauric acid (Fig. 8). When the NPs were irradiated by 808-nm laser, the temperature increased by more than 60 °C, which was enough heat to kill cancer cells, and simultaneously trigger release of AIPH via phase transition of lauric acid. Since AIPH causes cytotoxic radical in released state regardless of oxygen, this effect could occur in hypoxic condition. In addition, PDT and PTT effects were exhibited by using a single of laser in this study. In cytotoxicity assay, AIPH-loaded NPs showed a toxic effect with over 90% cell death, irrespective of oxygen concentration under laser irradiation. The authors intravenously injected these NPs into HepG2 tumor-bearing mice, and the NPs suppressed the tumor by 99.6%. Without AIPH, the tumor was suppressed by only 65.8%.

Recently, microalgae have been suggested as a solution to relieve hypoxia because microalgae enable greatly expeditious photocatalyzed oxygen generation through oxygenic photosynthesis process. Microalgae have been generally employed in diverse fields such as nutrition, bioremediation, and biofuel, which utilize their photosynthetic ability. In particular, chlorella vulgaris of unicellular microalgae has many advantages including abundant sources, low price, homogeneous structure, and lack of side effects. For these reasons, a study using microalgae's photosynthetic ability for alleviating hypoxia has been reported by Zhou group [52]. The authors used red blood cell membrane (RBCM) to modify the surface of the microalgae to reduce the uptake by macrophages and metabolic clearance of the microalgae. They proposed a combination of radiotherapy and PDT therapy with three types of irradiation. The initial red-light irradiation caused oxygen generation by RBCM-microalgae. The second X-ray irradiation released chlorophyll from the RBCM-microalgae as well as having an RT effect. The third 650-nm laser irradiation was used for PDT effect of the released chlorophyll as a PS. They injected RBCM-microalgae into 4T1 breast tumor-bearing BALB/c mice via a tail vein, and then irradiated sequentially as described above. RBCM-algae showed enhanced suppression of tumor growth for 12 days while the groups that received X-ray irradiation alone and RBCM-algae with single RT or PDT experienced only partial tumor growth inhibition. These results demonstrated that microalgae can potentially overcome hypoxia while providing PDT effect for anti-cancer therapy.

Type 1 reactions in PDT can react directly with biomolecules by transferring protons or electrons to produce radicals, whereas in type 2 reactions the excited PS transfers energy to oxygen molecules. For this reason, type 2 reaction is more affected by oxygen concentration than type 1. It means that type 1 reaction-based PDT could be effective in hypoxic tumor cell [53]. Recently, Zhang group synthesized tetrafluorophenyl bacteriochlorin as PS, which can generate oxygen-independent ROS through type 1 reaction [54]. They synthesized nanogels by crosslinking the PS with PEG to improve hydrophilicity and biodistribution. Then, they treated the nanogels to 4T1 cells and irradiated them with 750 nm-laser to activate the PS. As a result, both oxygen-dependent ROS (type 2 reaction) and oxygen-independent ROS (type 1 reaction) were generated by the nanogel in 4T1 cells. Moreover, when these nanogel-treated 4T1 cells were irradiated with a laser under hypoxia, the tumor cell killing effect was similar with that in normoxic condition. It demonstrates that the nanogel is a suitable agent to kill hypoxic tumor cells through type 1 reaction. Furthermore, they performed PDT in 4T1 tumor-bearing mice after i. v. injection of the nanogels by irradiating 750 nm-laser to tumor region. The nanogels significantly inhibited the growth of 4T1 tumors in different with the control groups.

Many attempts have been made to overcome hypoxia using oxygen carriers. However, they often suffer from barriers such as oxygen spillage, unavoidably leading to temporal hypoxia alleviation. Abundant oxygen may increase cancer cell proliferation and suppress cell systemic death. Therefore, instead of the oxygen carriers, the Kim and Peng group developed a method to alleviate hypoxia by reducing the amount of oxygen consumed during PDT [55]. The authors argued that cellular respiration such as the mitochondria-related oxidative phosphorylation process decisively contributes to intracellular oxygen consumption. To decrease cell respiration, they used tamoxifen, an antiestrogenic drug that can disturb the cell energy metabolism process by affecting the complex I in mitochondria electron transport chain. Type-1 PDT, which has lower oxygen dependence than type-2, can provide efficient PDT in a hypoxic environment. They synthesized a sulfur-substituted Nile blue analog as a PS for type-1 PDT and conjugated it with tamoxifen via click chemistry. The resulting unimolecular photodynamic O_2_-economizer was injected into 4T1 murine mammary tumor-bearing BALB/c mice via tail vein, and tumor sites were exposed to 660-nm laser irradiation. It showed 96% inhibition in tumor growth, which is highly efficient compared to the control group, demonstrating the importance of reducing oxygen consumption as well as supplying oxygen for PDT.

Another example of overcoming hypoxia using perfluorocarbon (PFC) was introduced by the Fan group [56]. The authors conjugated pyropheophorbide a (Ppa) and PFC to acetylated hyaluronic acid (Ac-HA) polymer via carbodiimide reaction between carboxyl and hydroxyl groups. Owing to the hydrophobicity of conjugated PFC and Ppa, the resulting Ac-HA-PFC-Ppa polymer forms amphiphilic micelle structure through self-assembly. Ac-HA facilitates tumor cell targeting of NPs due to binding and uptake via HA receptors and is decomposed by intracellular HAase after endocytosis. Because PFCs have weak intermolecular cohesive forces, it is possible for oxygen molecules to be inserted. Moreover, the PFCs have superb oxygen affinity, with high oxygen solubility and diffusivity. For these reasons, PFCs can carry a high content of oxygen as oxygen carriers. Consequently, tumor hypoxia is alleviated by the released PFCs. A self-assembled micelle was injected in OM431 uveal melanoma tumor-bearing BALB/c nude mice via a tail vein. After 21 days, the self-assembled micelles had significantly reduced tumor volume and weight more than the group without PFC. Based on these results, this research demonstrated that the simple oxygen self-carrying micelle structure could overcome hypoxia resistance of PDT.

### 4.2 Fenton reaction

ROS can be divided into a radical group (hydroxyl radical and superoxide) and a non-radical group (H_2_O_2_). Generally, H_2_O_2_ is abundant in cancer cells and relatively stable compared to other ROS molecules. It can penetrate cancer cell membranes and be changed into hydroxyl radicals in response to ferric or ferrous ions in cytosol via the Fenton reaction and Fenton-like reactions [57]. The hydroxyl radical is highly reactive and causes damage to cancer cells by reacting quickly with various biomolecules, including phospholipids, amino acids, and nucleic acids. Due to the high level of H_2_O_2_ in cancer cells, delivery of excess ferric ion to cancer cells could induce cell death by hydroxyl radicals produced by the Fenton reaction. Fe^3+^ can decompose H_2_O_2_ to produce O_2_, and in cancer cells, the reaction is more active because H_2_O_2_ level is higher than that in normal cells. For this reason, endogenous hypoxia can be relieved by Fenton reaction and many papers were already proved that by investigating the decrease of HIF-1 alpha or using hypoxia probe [58,59].

**Fig. 9:**
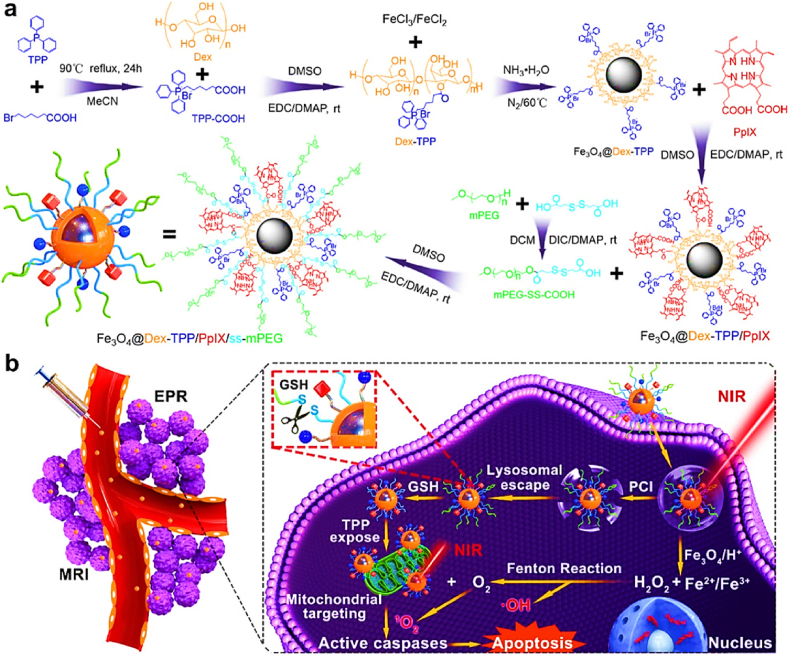
Fenton reaction-based photodynamic therapy with multifunctional magnetic nanoparticles. Adapted from Ref. [60]. Copyright 2019 American Chemical Society.

For a combination of PDT and Fenton reaction, Hou et al. developed dextran-grafted iron oxide NPs containing TPP and protoporphyrin IX (PpIX) for mitochondria targeting and PDT [60]. The resulting NPs showed efficient ROS generation and phototoxicity (IC50 8.257 μg/ml) compared to that of free form PS (IC50, 11.599 μg/ml) in 4T1 cells (Fig. 9). This means that iron oxide could improve the therapeutic efficacy of PDT via the Fenton reaction. When the surface of NPs was coated with glutathione-responsive disulfide PEG, the IC50 value further decreased to 2.698 μg/ml because PEG on the NP surface was detached and TPP was exposed to surface, allowing NPs to target mitochondria more efficiently. The NPs were also suitable for T2 magnetic resonance imaging, and they showed desirable tumor suppression for 14 days after intravenous injection into 4T1 tumor-bearing BALB/c mice. Another example of combinational PDT with Fenton reaction was introduced by the Hyeon group in 2017 [59]. They designed manganese ferrite NP-anchored mesoporous silica NP and Ce6 was loaded into the NP. Manganese ferrite NP has catalytic effect to decompose hydrogen peroxide to oxygen through Fenton reaction. To evaluate the oxygen production, they treated the NP to U-87 MG cells under hypoxia. As a result, the hypoxia marker, HIF-1a, was gradually decreased in a concentration-dependent manner of the NP. When PDT was performed on U-87 MG cells under hypoxic condition, free Ce6 only killed about 20% of the tumor cells, whereas the NP almost completely killed them due to the oxygen production through Fenton reaction. They intravenously injected the NP into U-87 MG tumor-bearing BALB/c nude mice. Oxygen level within tumor increased from 1.5% to 12.6%, which can enhance PDT efficacy. Furthermore, the researchers irradiated 670 nm-laser 24 h after i. v. injection of the NPs into the U-87 MG tumor-bearing mice to evaluate *in vivo* therapeutic efficacy. The NPs inhibited tumor growth successfully, but NPs without ferrite and manganese were less effective due to insufficient oxygen.

### 4.3 Combination with other drugs

A combination of PDT and immunotherapy has gained significant attention, because immunotherapy can make up for the low efficacy of PDT induced by hypoxia through enhancing inhibitory immune reaction on immune-suppressed tumor. In particular, targeting the immune checkpoint in tumor microenvironment relieves immune toleration of tumor and enhances PDT-induced immunity. Xing et al. suggested hypoxia-relieving NPs composed of fluorinated polymer, Ce6, and NLG919 [61]. NLG919 inhibits indoleamine 2,3-dioxygenase, which interrupts the differentiation of effector T cells and promotes that of regulatory T cells. As a result, the infiltration of CD4^+^ and CD8^+^ T cells increased significantly in the 4T1 tumor of BALB/c mice, leading to reinforcement of PDT-induced immunity. In addition, the oxygen-carrying fluorinated polymer supplied oxygen to the tumor, which helped with alleviation of hypoxia. The combination of these effects and PDT inhibited tumor growth up to 80.2% after intratumoral injection of NPs. Abscopal tumors also could be suppressed due to systemic immunity caused by NLG-919. These outcomes demonstrated that immunotherapy enhances PDT efficacy sufficiently to treat residual and metastatic tumors.

**Fig. 10:**
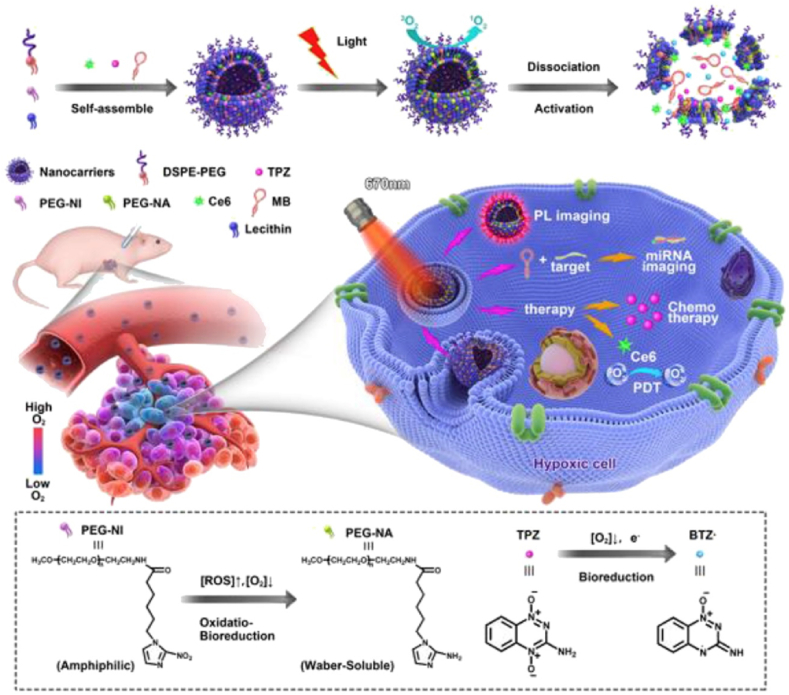
Combinational photodynamic therapy with tirapazamine, a hypoxia-activatable chemical drug. Adapted from Ref. [62]. Copyright 2018 Elsevier.

Tirapazamine (TPZ) is a hypoxia-responsive anticancer prodrug. It is converted to a more toxic form, benzotriazinyl radical, in low-oxygen environments such as tumor tissue. Treatment with TPZ only is not enough to destroy tumor tissue completely, particularly around oxygen-rich sites in the tumor. Therefore, Zhang et al. introduced a combination study of PDT and TPZ promoting synergistic antitumor effects by hypoxia-induced activation of TPZ [62]. They developed a hypoxia-reactive liposome containing amphiphilic 2-nitroimidazole (NI)-PEG, TPZ, gene probe, and Ce6 as PS (Fig. 10). ROS generation from Ce6 under a 670-nm laser irradiation consumed oxygen in the tumor tissue, resulting in severe hypoxia in the tumor tissue. This combination of high ROS and low-oxygen environment promoted reduction of NI group and changed PEG-NI from amphiphilic into hydrophilic, leading to disassembly of NPs. In addition, released TPZ was further activated in the hypoxic environment, damaging the tumor cells and DNA more efficiently. In *vitro* cytotoxicity tests on MCF-7 cells, these NPs showed effective tumor killing effect up to 80% with laser irradiation, while there was no change in free TPZ-treated group. After intravenous injection of NPs into MCF-7-bearing BALB/c nude mice, the fluorescence signal of Ce6 increased dramatically in the tumor site, in contrast to other main organs 12 h post-injection. Furthermore, during *in vivo* PDT, tumor growth was inhibited after laser irradiation and tumor tissue was almost eliminated 14 days post-injection of these NPs showing synergetic combination of TPZ and PDT.

Another unfavorable situation in tumor microenvironment is immunosuppressive condition such as PD-1/PD-L1 expression. Generally, PDT enhances secretion of interferon gamma to promote antitumor effect of T cells, but it may increase PD-L1 on tumor cell membrane, which decrease PDT-induced immune responses. In this point of view, it could be meaningful story that reduction of PD-L1 on tumor cell membrane during PDT. Xiong et al. introduced a combination study between metformin, PD-L1 down-regulator, and IR775, an ICG analog PS [63]. They encapsulated metformin and IR775 into liposome and its size was about 153 nm. When the liposomes were intravenously injected into MB49 tumor-bearing C57BL/6 mice, PD-L1 expression in tumor decreased to about 30% compared to control group. The liposome alleviated the immunosuppressive microenvironment of the tumor tissue and increased T cell infiltration by two times compared to liposomes loaded with only IR775. The researchers injected the liposomes into MB49 tumor-bearing mice and irradiated with 785 nm laser to investigate PDT efficacy *in vivo*. The liposome almost eliminated the tumor, and the tumor weight after treatment was about six times lighter than that of the control group. On the other hand, metformin-free liposomes only slowed insufficient tumor suppression. This means that the reversal of the immunosuppressive state of the tumor tissue by metformin can promote PDT-induced immune response.

## 5 Conclusion and perspective

In summary, we have introduced recent studies based on NPs that overcome obstacles of PDT in tumor tissue and organized them in Table 1. Insufficient supply of pivotal factors including PS, light, and oxygen highly reduces the therapeutic efficacy of PDT. Based on recent progress in the combination of nano- and biotechnology, various kinds of NPs have been developed for PDT, and they showed promising potential to overcome these obstacles. Currently, many of these ideas are at the proof-of-concept level, and more efforts are needed to get closer to commercialization and clinical application. Considering the explosive attention on NPs, the number of NP formulations currently approved by FDA and being used in clinical practice is less than expected. It may originate from two problems, safety and economic issues. About safety issue, FDA-approved molecules or body components need to be preferred in designing NPs, and toxic materials need to be excluded at the same time even though they did not show acute toxicity in cell test [64]. Moreover, long synthetic or formulation steps are frequently considered to achieve multi-functions, but they are not advantageous for massive production and commercialization [65]. Physicochemical optimization for passive targeting, ligand modification for active targeting, and stimuli-responsive release achieved efficient PS delivery to tumor tissue. However, penetration of drugs into deep tumor tissue is an important issue in general drug delivery systems. NPs are larger than small-molecule drugs, so their penetration is more difficult than that of free drugs. Therefore, deep tissue penetration of PS-containing NPs needs to be considered carefully to obtain better therapeutic outcomes, and novel ideas including size-shrinkable NPs or ECM-penetrating NPs will be helpful [[66], [67], [68]]. Various trials using upconversion NPs, two-photon lasers, X-rays, and bioluminescence have provided clues for efficient light delivery to deep tissue. Many of these studies used metal NPs, which are not free from safety issues [69]. In particular, as we learned from the example of quantum dot in biomedical fields, the potential toxicity of inorganic upconversion NPs composed of heavy metals is a critical limitation and recent approaches with organic upconversion NPs is valuable [70,71]. Therefore, their potential toxicity needs to be considered seriously and favorable distribution, degradation, and excretion should be achieved [72]. Attempts to overcome oxygen deficiency in tumor tissue due to hypoxia have included artificial oxygen generation, Fenton reaction, and combination with hypoxia-related chemical drugs. Besides their promising results, combination with recent approaches to normalize tumor vasculatures and reduce hypoxia itself will also be promising for efficient tumor therapy by PDT. Although the introduced studies are in the early stages, they have led to significant progress over traditional PDT studies by focusing on critical hurdles. Therefore, we expect the results of these trials to maximize the effectiveness of clinical treatment with PDT in the future.

**Table 1:** Summary of recently developed NPs to overcome the obstacles of current photodynamic therapy in tumor. Abbreviation: Nanoparticle (NP), Polyethylene glycol (PEG), Upconversion nanparticle (UCNP), Photosensitizer (PS).

Obstacles to overcome	NP type	Strategy	Tumor cell (Mouse)	Injection	Irradiation	Year	ref
PS delivery	Human serum albumin NP	Ultrasound-based cell membrane perturbation to enhance tumor accumulation	4T1 (BALB/c)	i.v	635 nm laser	2020	[22]
	PEGylated polymeric micelle	Extracellular matrix degradation by hyaluronidase to enhance penetration	4T1 (BALB/c)	i.v.	660 nm laser	2016	[23]
	Crosslinked human serum albumin	Hypoxia-responsive degradation of azobenzene and NP dissociation for deep tissue penetration	4T1 (BALB/c)	i.v.	660 nm laser	2019	[27]
	Block copolymer micelle	Singlet oxygen-responsive degradation of vinyldithioether and PS release to increase specificity	A375 (BALB/c nude)	i.v.	660 nm laser	2020	[28]
	Fucoidan-based nanogel	Binding between fucoidan and P-selectin on tumor endothelial cells	HT1080 (BALB/c nude)	i.v.	670 nm laser	2020	[30]
	Block copolymer micelle	pH-responsive PS release and mitochondrial targeting by TPP	HO8910 (Nu/Nu nude)	i.v.	660 nm laser	2019	[31]
	Polymer-coated micelle	Surface charge reversal in tumor tissue and mitochondrial targeting by TPP	CT26 (BALB/c)	i.v.	660 nm laser	2020	[32]
Light delivery	Polymer-grafted UCNP	pH-responsive dequenching of PS and PDT using upconverted light from UCNP	A549 (BALB/c nude)	i.t.	980 nm laser	2018	[36]
	Co-assembled structure with two UCNPs	Multi-step photoactivation and PDT using dual UCNPs	Cal-27 (BALB/c nude)	i.t.	808 nm and 980 nm laser	2019	[37]
	Hyperbranched polymeric micelle	Thermo-response and Two-photon-activated FRET to excite PS	Hela (BALB/c nude)	i.v.	800 nm two photon laser	2016	[39]
	Phospholipid-polymer NP	Two-photon laser-based PDT for better penetration	Hela (BALB/c nude)	i.t.	800 nm two photon laser	2017	[40]
	Gd_2_(WO_4_)_3_:Tb scintillator	Synergistic radiotherapy and X-ray-based PDT by nanoscintillators	4T1 (BALB/c)	i.v.	X-ray of 6 Gy	2019	[43]
	Silicate nano-scintillator	Low dose X-ray-based PDT to minimize normal tissue toxicity	U87MG (BALB/c nude)	i.v.	X-ray of 1 Gy	2019	[44]
	PEG-dextran polymeric NP	Oxalate-based chemiluminescence for PDT to overcome laser penetration depth	HT29 (BALB/c nude)	i.v.	Chemiluminescence	2020	[46]
	Polymeric micelle	PDT with luminol-based chemiluminescence activated under oxidative conditions	A549 (BALB/c nude)	i.v.	Chemiluminescence	2019	[47]
	Poly (lactic-*co*-glycolic acid) NP	PDT using BRET between luciferase-based bioluminescence and PS	H22 (ICR)	i.t.	Bioluminescence	2018	[48]
Unfavorable tumor microenvironments	Bismuth selenide NP	Oxygen-independent PDT using radical generator	H22 (ICR)	i.v.	808 nm laser	2019	[51]
	Red blood cell membrane-modified microalgae	Oxygen enrichment in tumor and PDT by microalgae	4T1 (BALB/c)	i.v.	660 nm laser and X-ray of 2 Gy	2020	[52]
	Tamoxifen-PS conjugate	Intracellular oxygen enrichment by blocking mitochondria respiration	4T1 (BALB/c)	i.v.	660 nm laser	2020	[55]
	Hyaluronic acid- perfluorocarbon NP	Elevating intracellular oxygen level using perfluorocarbon	OM431 (BALB/c nude)	i.v.	660 nm laser	2020	[56]
	Dextran-grafted iron oxide NP	Elevating intracellular oxygen level by Fenton reaction	4T1 (BALB/c)	i.v.	637 nm laser	2019	[60]
	Ferrite NP-anchored meshporous silica NP	Elevating intracellular oxygen level by Fenton reaction	U-87 MG (BALB/c nude)	i.v	670 nm laser	2017	[59]
	Fluorinated polymer NP	Reinforcement of PDT-induced immunity by NLG919	4T1 (BALB/c)	i.t.	660 nm laser	2019	[61]
	PEGylated liposome	Synergy with hypoxia-activated prodrug, Tirapazamine	MCF-7 (BALB/c)	i.v.	670 nm laser	2018	[62]
	PEGylated liposome	Reduction of immunosuppressive PD-L1 on tumor cell membrane	MB49 (C57BL/6)	i.v	785 nm laser	2021	[63]

## Declaration of competing interest

The authors declare no conflict of interest.

## CRediT authorship contribution statement

**Donghyun Lee:** Conceptualization, Writing – review & editing. **Soonmin Kwon:** Writing – review & editing. **Seok-young Jang:** Writing – review & editing. **Eunyoung Park:** Writing – review & editing. **Yeeun Lee:** Writing – review & editing. **Heebeom Koo:** Conceptualization, Writing – review & editing, Supervision, Funding acquisition.
